# The ND250 indexes a visual–orthographic familiarity effect in visual word recognition: evidence from Chinese components

**DOI:** 10.3389/fpsyg.2026.1783812

**Published:** 2026-05-12

**Authors:** Ziyi Huang, Jinke Hao, Jianghua Han, Feng Gu

**Affiliations:** 1 Neurocognitive Laboratory for Linguistics and Semiotics, College of Literature and Journalism, Sichuan University, Chengdu, China; 2 Digital Convergence Laboratory of Chinese Cultural Inheritance and Global Communication, Sichuan University, Chengdu, China

**Keywords:** Chinese characters, Chinese components, ND250, visual word recognition, visual–orthographic familiarity

## Abstract

Reading pseudowords (or low-frequency words) typically elicits stronger brain activation than real words (or high-frequency words) in the ventral occipitotemporal cortex (vOT). The ND250 has been proposed as an event-related potential (ERP) component indexing this enhanced neural response. However, the neural basis of the ND250 and its functional role in visual word recognition remain unclear. Within the framework of the Interactive Account, the ND250 has been interpreted as a prediction error signal, reflecting phonological–semantic top-down influences on orthographic processing. Alternatively, it can be interpreted as a visual–orthographic familiarity effect, with greater neural responses to unfamiliar orthographic stimuli than to familiar ones. The present study employed Chinese components as stimuli to dissociate visual-form frequency from phonological and semantic associations. ERPs to Chinese components were recorded during an implicit reading task. The results showed that the ND250 effect was not modulated by the frequency with which an orthographic stimulus (i.e., a Chinese component) is associated with phonological and semantic information. Instead, it was selectively modulated by the frequency with which the component’s visual form appears in written text. These findings challenge the interpretation of the ND250 as a prediction error signal proposed by the Interactive Account. Rather, they suggest that, under implicit task conditions, the ND250 is more consistent with the visual–orthographic familiarity account. By dissociating visual-form frequency from phonological and semantic associations, the present study advances our understanding of the neural basis of the ND250 and provides new insights into early visual–orthographic processing in visual word recognition.

## 1 Introduction

The ability to read is indispensable in modern society, and event-related potentials (ERPs) provide a powerful approach for investigating the neural mechanisms underlying reading. A central question in reading research is how a string of visual symbols is mapped onto its lexical identity within the mental lexicon. A number of ERP studies have sought to characterize the neural activity associated with orthographic processing. A recently named ERP component, the ND250 (Liu et al., 2025; Hu et al., 2026), has been proposed to reflect this stage. The ND250 is characterized by a negative-going ERP deflection elicited by pseudowords relative to real words—or by low-frequency words relative to high-frequency words—maximally distributed over occipitotemporal scalp regions approximately 250 ms after word onset. Source localization results consistently indicate that the ND250 originates from the ventral occipitotemporal cortex (vOT) (Zhang et al., 2023; Liu et al., 2025). Before being labeled as the ND250, this effect was usually referred to as the “250-ms ERP difference” and has been consistently observed across multiple languages, including English (Hauk et al., 2006; Hauk et al., 2012; Kim and Strakova, 2012; Dufau et al., 2015), French (Rosazza et al., 2009; Strijkers et al., 2015), Spanish (Vergara-Martinez et al., 2021), and Chinese (Wang and Maurer, 2017; Yum et al., 2018; Huang et al., 2022; Yu et al., 2022; Huang et al., 2023; Zhang et al., 2023). Despite its robust and cross-linguistic presence, however, the neural basis of the ND250 and its functional role in visual word recognition are not yet well understood.

Because the direction of ERP deflection does not directly reflect increases or decreases in neural activation, the functional interpretation of the ND250 has been debated. Recent evidence favors the view that the ND250 reflects enhanced neural responses to pseudowords relative to real words (or to low-frequency words relative to high-frequency words), rather than the reverse pattern (Liu et al., 2025). This interpretation is consistent with numerous fMRI/PET studies that have reported increased brain activation in the vOT when processing pseudowords compared to real words, or low-frequency words compared to high-frequency words (e.g., Xu et al., 2001; Fiebach et al., 2002; Chee et al., 2003; Kuo et al., 2003; Mechelli et al., 2003; Joubert et al., 2004; Carreiras et al., 2006; Hauk et al., 2008; Carreiras et al., 2009; Graves et al., 2010; Wang et al., 2011). Converging evidence from an intracranial recording study further showed greater neural activity in the vOT for words relative to nonwords, as well as for low-frequency words relative to high-frequency words, around 250 ms after word onset (Woolnough et al., 2021). The increased neural activity, including ND250, can be effectively interpreted as a prediction error signal within the framework of the Interactive Account (Price and Devlin, 2011). This framework proposes that the vOT does not operate as a purely bottom-up orthographic processor, but rather as an interface that integrates bottom-up visual input with top-down predictions from higher-order phonological and semantic representations. When a visual stimulus has strong pre-existing phonological-semantic associations, these higher-order representations can generate stronger and more precise predictions about the expected orthographic input. As a result, the mismatch between top-down prediction and bottom-up visual input is reduced, leading to a smaller prediction error and thus reduced neural activation. In contrast, when a stimulus has weak phonological-semantic associations, top-down predictions are weaker or less specific, resulting in a larger mismatch between prediction and sensory input. This larger mismatch gives rise to a larger prediction error and consequently enhanced neural activation.

However, the ND250 can also be alternatively interpreted as reflecting a visual–orthographic familiarity effect. Several fMRI studies have used pseudohomophones to dissociate the effects of visual–orthographic familiarity and phonological–semantic familiarity on neural activation during visual word recognition (Kronbichler et al., 2004; Kronbichler et al., 2007; Bruno et al., 2008). These studies consistently reported that activity in the vOT is strongly modulated by visual–orthographic familiarity, but not by phonological–semantic familiarity, with greater neural activation elicited by orthographically unfamiliar stimuli than by familiar ones. Converging evidence comes from a study by Twomey et al. (2013), who investigated Japanese visual word recognition—an ideal test case for disentangling these two factors because Japanese words can be written either in Kanji (logographic script) or Hiragana (syllabic script). Their results likewise revealed a robust effect of visual–orthographic familiarity on vOT activity, with no reliable contribution from phonological–semantic familiarity. Further support comes from ERP research, which shows that electrophysiological responses around 200 ms are primarily sensitive to orthographic properties and are not reliably modulated by phonological or semantic factors (Zhang et al., 2012; Hu et al., 2025). Taken together, these findings suggest that visual–orthographic familiarity may underlie the elicitation of the ND250, independently of phonological and semantic influences.

Understanding the functional basis of the ND250 is crucial for clarifying the mechanisms underlying visual word recognition. However, previous studies have typically relied on manipulations in which visual–orthographic familiarity and phonological–semantic associations are confounded, making it difficult to adjudicate between competing accounts (i.e., the Interactive Account vs. the visual–orthographic familiarity account). Specifically, real words and high-frequency words are generally both orthographically familiar and strongly associated with phonological and semantic representations, whereas pseudowords and low-frequency words are both orthographically less familiar and have weaker or absent phonological–semantic associations. As a result, it remains unclear whether ND250 effects are driven by visual–orthographic familiarity or by top-down phonological–semantic influences. To address this limitation, the present study tests these competing explanations by using Chinese components as stimuli, which allow visual–orthographic familiarity and phonological–semantic associations to be dissociated. For instance, the Chinese component “禾” is highly orthographically familiar because it appears frequently as a constituent of many characters, but its pronunciation and meaning are typically not accessed from these characters during normal reading, and thus its orthographic–phonological–semantic associations are relatively weak.

**Figure 1:**
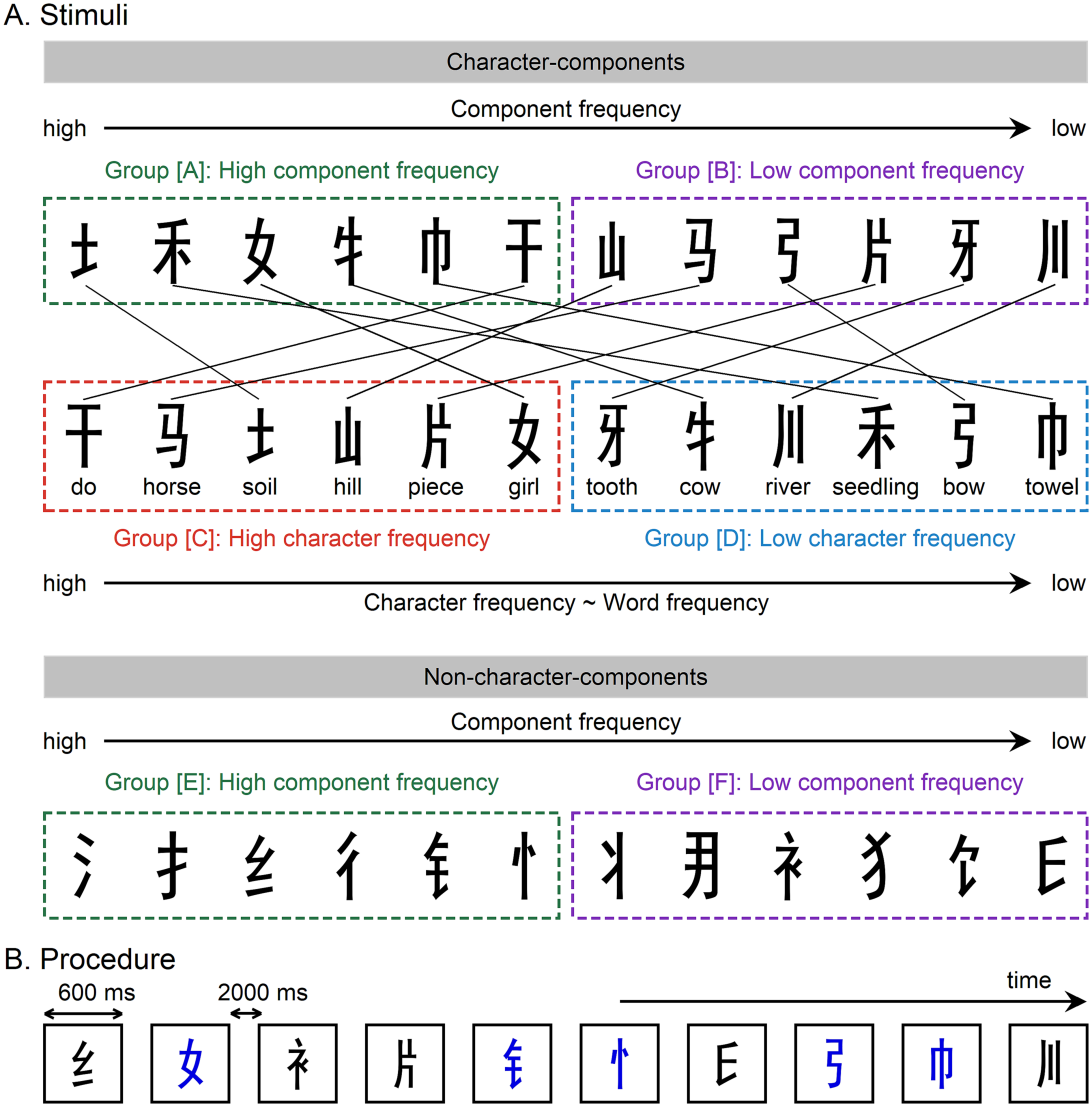
Stimuli and procedure. **(A)** Twelve character-components and 12 non-character-components were used as stimuli. English translations for these character-components are provided. The 12 character-components are sorted by either component frequency or character frequency from high to low, while the non-character-components are sorted by component frequency from high to low. **(B)** The 24 components were pseudo-randomly presented at the center of a monitor. Each stimulus was displayed for 600 ms, followed by a 2,000 ms inter-stimulus interval. Participants were instructed to judge the color of each stimulus (black or blue).

Chinese is a logographic writing system in which characters primarily convey meaning rather than pronunciation. Almost every Chinese character (*hanzi*, or *zi*) corresponds to a meaningful morpheme with a syllabic pronunciation. Each character is composed of one or more components, also referred to as radicals. Some components possess their own pronunciation and morphemic meaning and can therefore function as independent characters. Others are unpronounceable and lack morphemic meaning, and can only occur in combination with other components to form a meaningful and pronounceable character. The former are referred to as *character-formation components*, whereas the latter are termed *character non-formation components*. For simplicity, these are hereafter referred to as *character-components* and *non-character-components*, respectively. In the present study, 12 character-components and 12 non-character-components were used as stimuli (Figure 1A). All stimuli were intermixed and presented individually in a pseudo-randomized sequence (Figure 1B). Participants performed an implicit reading task in the form of a color-decision task, in which they were required to identify the color of each stimulus. This task was adopted because previous studies have demonstrated that the ND250 reflects obligatory and automatic orthographic processing, with comparable amplitudes observed in both explicit and implicit reading tasks (Yu et al., 2022; Zhang et al., 2023).

The 12 character-components used in the present study can be sorted by both component frequency and character frequency from high to low (Figure 1A). Component frequency is measured by the number of occurrences of a particular component per 1 million characters in written text. It reflects the frequency of visual exposure to the component itself, independent of any phonological or semantic associations, and serves as an index of visual-orthographic familiarity. In contrast, character frequency represents the number of occurrences of a particular character per 1 million characters in written text. This measure indicates how frequently a pronounceable and meaningful character is encountered in context. For example, the component “马” can function both as a sublexical element within a character (e.g., “骄”) and as an independent character meaning “horse.” In this case, its component frequency reflects how often it appears in written language, whether as a sublexical element or as an independent character. In contrast, its character frequency reflects how often the character “马” itself appears in written language, thereby indexing how frequently it is associated with stable phonological and semantic information during reading. Component frequency is analogous to letter frequency in English. Letter frequency measures how often a letter appears in written text. For instance, the letter frequency of the letter “a” is determined by its total occurrences, whether as an independent word (“a”) or as part of another word (e.g., the “a” in “has”). In contrast, character frequency corresponds to word frequency in English. Word frequency reflects how often a specific word appears in text. For example, the word frequency of “a” is calculated based solely on how often the word “a” occurs, excluding instances where “a” is part of another word.

The logic of the present design is based on dissociating visual–orthographic familiarity from phonological–semantic associations and examining their respective contributions to the ND250. According to the Interactive Account, if the ND250 reflects a prediction error signal driven by top-down phonological and semantic influences, it should be sensitive to differences in phonological–semantic associations. This prediction is tested by comparing high character-frequency character components (group [C] in Figure 1A) with low character-frequency character components (group [D]), while controlling for component frequency. Because components in the high character-frequency group are more frequently associated with stable phonological and semantic representations during reading, this contrast selectively manipulates the strength of phonological–semantic associations. The Interactive Account therefore predicts that low character-frequency components (group [D]) should elicit a more negative ERP deflection over occipitotemporal scalp regions (i.e., a larger ND250 amplitude) than high character-frequency components (group [C]). In contrast, the visual–orthographic familiarity account predicts that the ND250 should be driven by differences in visual–orthographic familiarity. This prediction is tested by comparing high component-frequency character components (group [A]) with low component-frequency character components (group [B]), while controlling for character frequency. Because components in the high component-frequency group appear more frequently in written text, this contrast selectively manipulates visual–orthographic familiarity. The visual–orthographic familiarity account therefore predicts that low component-frequency components (group [B]) should elicit a more negative ERP deflection over occipitotemporal scalp regions (i.e., larger ND250 amplitude) than high component-frequency components (group [A]), whereas the Interactive Account does not. Moreover, this prediction is further tested using non-character components. The comparison between high component-frequency non-character components (group [E]) and low component-frequency non-character components (group [F]) isolates visual–orthographic familiarity in the absence of stable phonological and semantic representations. In this case, the visual–orthographic familiarity account predicts a directional effect: low component-frequency non-character components (group [F]) should elicit a more negative ERP deflection over occipitotemporal scalp regions (i.e., a larger ND250 amplitude) than high component-frequency non-character components (group [E]), whereas the Interactive Account predicts no reliable effect.

## 2 Materials and methods

### 2.1 Participants

Thirty-eight native Chinese readers (23 females, 15 males; aged 18–25 years, *M* = 20.42, *SD* = 2.11) voluntarily participated in the experiment. Data from two additional participants were excluded due to excessive artifacts in the EEG recordings. Sample size requirements were determined via an *a priori* power analysis using G*Power (version 3.1). For a paired-samples *t* test with a medium effect size (Cohen’s *d* = 0.5), 80% statistical power, and a two-tailed *α* level of 0.05, the minimum required sample size was 34 participants.

All participants were students at Sichuan University and reported normal or corrected-to-normal vision. None reported a history of reading disabilities or neurological disorders. Handedness was assessed using the Edinburgh Handedness Inventory (Oldfield, 1971), confirming that all participants were right-handed. Written informed consent was obtained from each participant prior to the experiment, and all procedures were approved by the Ethics Committee of the College of Literature and Journalism at Sichuan University.

### 2.2 Stimuli

Twenty-four Chinese components were used as stimuli (see Figure 1A and Table 1), comprising 12 character-components and 12 non-character-components. Notably, the component “彳” is an ancient Chinese character meaning “taking small steps.” It is not used in modern Chinese and does not appear in Chinese lexical databases (e.g., Cai and Brysbaert, 2010; Sun et al., 2018), effectively giving it a character frequency of zero. Most skilled Chinese readers, particularly the participants in this study, are unfamiliar with this specific character, lacking knowledge of both its pronunciation and semantic meaning. Therefore, it is classified as a non-character component. The linguistic properties of these components are listed in Table 1, including the number of strokes (an indicator of visual complexity), component frequency, character frequency, pronunciation (in Chinese pinyin), and character or morphemic meanings.

**Table 1:** Stimulus properties.

Character-components:	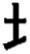	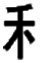	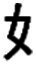	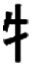	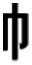	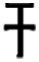	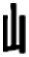	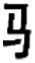	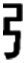	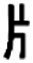	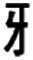	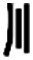
Strokes	3	5	3	4	3	3	3	3	3	4	4	3
Component frequency	18,588.1	9,253.1	7,957.3	2,988.4	2,856.8	1,986.1	1,871.1	1,602.1	1,225.7	401.9	372.4	271.3
Character frequency	1,002.6	30.9	266.9	160.6	12.4	1,633.5	889	1,240.6	14.8	519.4	171	98.2
Pronunciation	tǔ	hé	nǚ	niú	jīn	gàn	shān	mǎ	gōng	piàn	yá	chuān
Meaning	soil	seedling	girl	cow	towel	do	hill	horse	bow	piece	tooth	river

Non-character-component	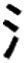	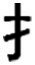	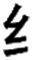	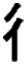	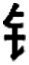	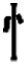	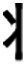	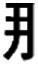	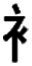	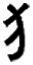	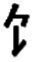	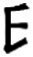
Strokes	3	3	3	3	5	3	3	4	5	3	3	3
Component frequency	13,329.6	12,322.2	10,180.4	4,626.7	2,617.2	2,570.5	1,246.1	750.9	731.0	598.9	343.2	93.6
Character frequency	0	0	0	0	0	0	0	0	0	0	0	0
Pronunciation	NA	NA	NA	NA	NA	NA	NA	NA	NA	NA	NA	NA
Meaning	NA	NA	NA	NA	NA	NA	NA	NA	NA	NA	NA	NA

Strokes refers to the number of strokes in a component. Component frequency refers to the number of times a component is found in 1 million characters. Character frequency refers to the number of times a character is found in 1 million characters. Both component and character frequencies were obtained from the Dictionary of Chinese Character Information (1988). Pronunciations (in Chinese pinyin) and meanings of the character-components are provided. NA indicates not available.

In Chinese, components in up–down structured characters are typically short and wide (e.g., “女” in “要”), whereas those in left–right structured characters are generally tall and slender (e.g., “女” in “好”). In the present study, all components used as stimuli were extracted from left–right structured characters and therefore appeared in a tall and slender form (see Figure 1), ensuring uniformity in visual shape across all stimuli. The mean log-transformed character and component frequencies for each stimulus group (see Figure 1A for stimulus grouping) are reported in Supplementary Table S1. Component frequency differed significantly between group [A] and group [B], *t*(10) = 4.043, *p* = 0.002 (two-tailed), whereas character frequency was well matched between the two groups, *t*(10) = 0.269, *p* = 0.793 (two-tailed). In contrast, character frequency differed significantly between group [C] and group [D], *t*(10) = 5.015, *p* = 0.001 (two-tailed), while component frequency was matched, *t*(10) = 0.770, *p* = 0.459 (two-tailed). Finally, group [E] and group [F] also differed significantly in component frequency, *t*(10) = 5.231, *p* < 0.001 (two-tailed).

### 2.3 Procedure

Participants were seated in a sound-attenuated, dimly lit room at a viewing distance of approximately 135 cm from the screen. Before the formal experiment, each participant completed a short practice session to become familiar with the task.

A color-decision task, commonly used as an implicit reading paradigm, was adopted in the present study. In each trial, a single stimulus was presented at the center of the screen in Heiti font against a white background (see Figure 1B). Each stimulus subtended approximately 1.7° of visual angle (about 4 cm in width) and was displayed for 600 ms, followed by a 2,000 ms inter-stimulus interval. Participants were instructed to indicate the color of each stimulus (black or blue) as quickly and accurately as possible by pressing two adjacent keys with their right index and middle fingers.

The experiment consisted of four blocks. Within each block, each of the 24 components was presented 10 times (five in black and five in blue), resulting in a total of 960 trials across the experiment, with each component appearing 40 times in total. Previous research has shown that this level of stimulus repetition (40 presentations) does not significantly attenuate ND250 amplitude (Yu et al., 2022). Stimuli were presented in a pseudo-random order with the following constraints: (1) the same component did not appear consecutively; (2) no more than three stimuli of the same color were presented consecutively; and (3) no more than three stimuli of the same characterhood (character vs. non-character) were presented consecutively. Short breaks were provided between blocks to reduce fatigue. Stimulus presentation and behavioral data collection were controlled using E-Prime (version 3.0). The entire session lasted approximately 1.5 h.

### 2.4 EEG recording

EEG activity was recorded with a 64-channel elastic cap fitted with Ag/AgCl electrodes and amplified using a SynAmps 2 system (NeuroScan, Charlotte, NC, USA). The electrode montage followed the extended International 10–20 convention. The nasal tip served as the reference, and the ground electrode was placed between FPz and Fz. Additionally, vertical electrooculogram (VEOG) activity was recorded using two electrodes placed superior and inferior to the left eye. The impedance was kept below 5 kΩ between any electrode and the reference electrode. Continuous EEG data (0.05–200 Hz) were sampled at 500 Hz.

### 2.5 Data analysis

#### 2.5.1 EEG preprocessing

For each participant, EEG data were processed through a standard preprocessing pipeline to derive ERPs. First, the continuous EEG was band-pass filtered between 0.1 and 25 Hz. Eye-blink–related ocular artifacts were then corrected using a regression-based method (Semlitsch et al., 1986). The filtered data were subsequently segmented into epochs ranging from 100 ms before to 500 ms after stimulus onset, followed by baseline correction using the 100-ms pre-stimulus interval. Epochs with voltage amplitudes exceeding ±50 μV at any unipolar electrode were excluded from further analysis. The remaining trials were averaged separately for each stimulus group (groups [A]–[F]; see Figure 1A for stimulus grouping). Finally, the averaged ERPs were re-referenced to the mean of the 64 unipolar electrodes.

#### 2.5.2 Statistical analysis of ND250 elicitation

Given that the timing and spatial distribution of the ND250 may vary as a function of stimulus type (e.g., Yu et al., 2022; Huang et al., 2023), regions of interest (ROIs) and time windows could not be defined with sufficient precision based on *a priori* knowledge. Therefore, the present study adopted a data-driven approach to assess whether a significant ND250 was elicited by one stimulus group relative to another. Specifically, ERPs elicited by the two groups were compared using repeated-measures, two-tailed *t* tests at every sampling point (2 ms resolution) across all 64 electrodes, within a time window ranging from 50 to 500 ms post-stimulus onset. This procedure resulted in a large number of statistical comparisons (14,400 tests), substantially increasing the risk of Type I errors. To control for multiple comparisons, the false discovery rate (FDR) was controlled using the Benjamini–Hochberg procedure (Benjamini and Hochberg, 1995), ensuring that the expected proportion of false positives remained within an acceptable range. In addition, because three group-wise comparisons were conducted (i.e., group [A] vs. [B], group [C] vs. [D], and group [E] vs. [F]), we applied a more stringent FDR threshold (*q* = 0.0167; 0.05/3) to provide a conservative control across these planned contrasts. Mass univariate ERP analyses were conducted using the Mass Univariate ERP Toolbox (Groppe et al., 2011).

## 3 Results

### 3.1 Behavioral results

The mean accuracy rates and response times for each stimulus group are reported in Supplementary Table S2. Accuracy was uniformly high across all groups (mean > 96%), indicating that participants performed the task accurately. Paired-samples *t* tests conducted on log-transformed response times revealed no significant differences between groups [A] and [B], [C] and [D], or [E] and [F] (*ps* > 0.058, two-tailed, uncorrected).

### 3.2 ERP results

**Figure 2:**
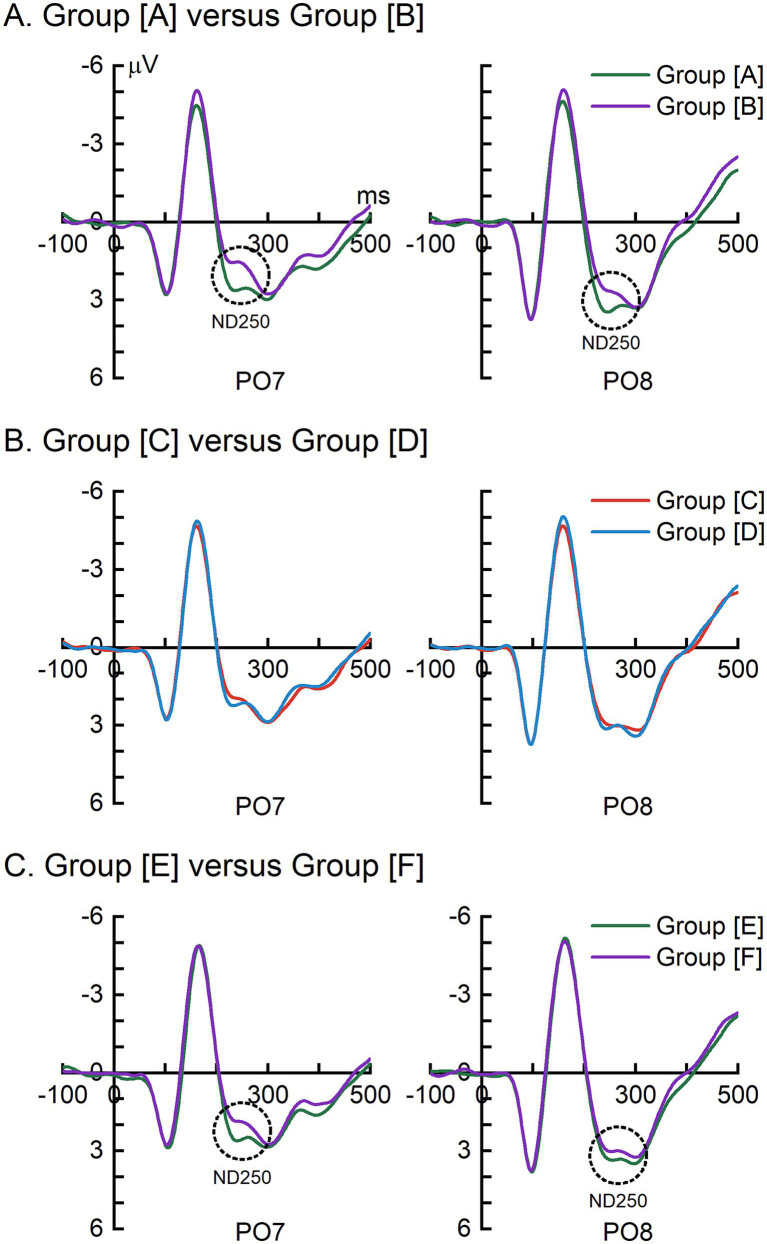
Grand-averaged ERPs recorded at electrodes PO7 and PO8. **(A)** Comparison of ERPs elicited by group [A] and group [B]. **(B)** Comparison of ERPs elicited by group [C] and group [D]. **(C)** Comparison of ERPs elicited by group [E] and group [F]. Dotted circles highlight the ND250 effect.

For each stimulus group, grand-averaged ERPs recorded at electrodes PO7 and PO8, where the ND250 typically shows the largest amplitude (Yu et al., 2022; Zhang et al., 2023), are presented in Figure 2. A clear ND250 effect (highlighted by dotted circles) was observed for the comparisons between group [A] and group [B], as well as between group [E] and group [F], but not between group [C] and group [D].

**Figure 3:**
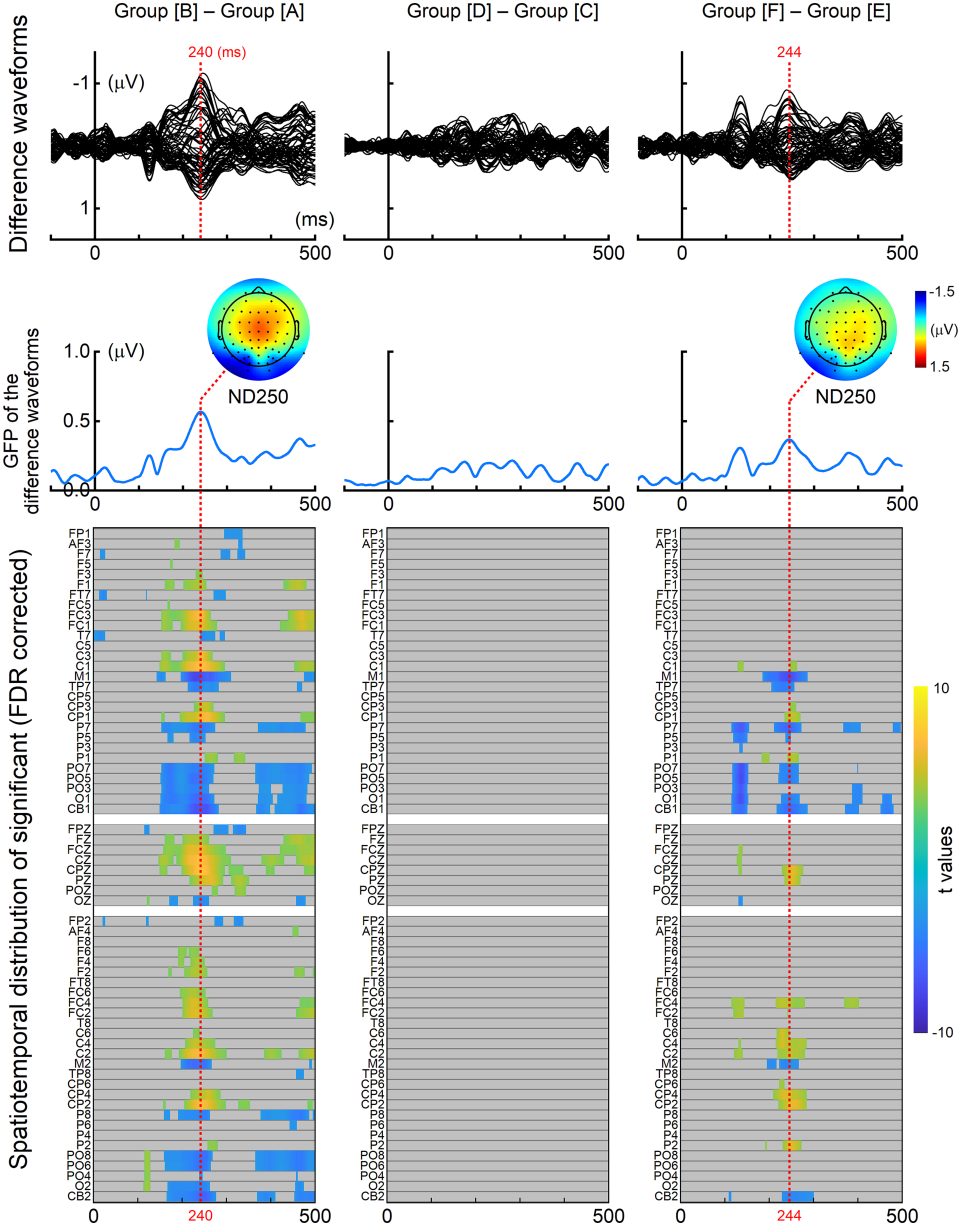
ERP difference between stimulus groups. ERPs elicited by one stimulus group were subtracted from those elicited by the other group at each of the 64 recording electrodes. The resulting grand-averaged difference waveforms are shown in the top panels. The global field power (GFP) of the difference waveforms for each comparison is displayed in the middle panels. Repeated-measures, two-tailed *t* tests were conducted at each time point between 50 and 500 ms post-stimulus onset. The resulting spatiotemporal distribution of significant effects, corrected for multiple comparisons using the FDR procedure, is shown in the bottom panels. Non-significant time points are masked in gray, whereas significant time points are visualized using a heatmap, with color intensity representing the corresponding *t* values. Numerical *t*-values and corresponding effect size estimates (Cohen’s *d*) are provided in Supplementary material.

Figure 3 presents the results of the mass univariate ERP analyses across stimulus groups. Consistent with the ERP waveforms shown in Figure 2, significant ND250 effects were observed for the comparisons between group [A] and group [B], as well as between group [E] and group [F]. In contrast, no significant ND250 effect was detected for the comparison between group [C] and group [D]. In addition to the ND250 effects, significant ERP differences were also observed at earlier latencies (i.e., before 200 ms), which may be attributable to differences in low-level visual properties between the compared groups, as individual Chinese components differ in their visual features.

## 4 Discussion

The present study used Chinese components as stimuli to examine whether the ND250 is driven by the frequency with which an orthographic stimulus is associated with phonological and semantic information, as proposed by the Interactive Account, or by the frequency of visual exposure to its form in written text, as predicted by the visual–orthographic familiarity account. The results are consistent with the latter explanation. The implications of these findings are discussed below.

### 4.1 ND250 indexes a visual-orthographic familiarity effect

The present findings suggest that the ND250 does not reflect a prediction error signal arising from higher-order phonological and semantic influences on orthographic processing, as proposed by the Interactive Account. Instead, the ND250 appears to be driven by increased neural activation to visually less familiar stimuli relative to more familiar ones, consistent with a visual-orthographic familiarity effect. As outlined in the Introduction, numerous fMRI, PET, and intracranial recording studies have reported increased vOT activation for pseudowords relative to real words, or for low-frequency words relative to high-frequency words. If the neural responses observed in these studies reflect the same underlying processes indexed by the ND250, they may instead be more parsimoniously explained in terms of visual-orthographic familiarity. Consistent with this view, several fMRI studies have shown that vOT activity is modulated by visual–orthographic familiarity rather than by phonological or semantic familiarity (Kronbichler et al., 2004; Kronbichler et al., 2007; Bruno et al., 2008; Twomey et al., 2013).

Building on extensive evidence from visual object recognition research, we further propose that the ND250 may reflect a domain-general visual familiarity effect. A large body of research in visual object recognition has shown that unfamiliar objects elicit stronger neural responses than familiar objects in the inferior temporal (IT) cortex of both monkeys and humans—a region within the ventral visual processing stream that also encompasses areas involved in visual word recognition, such as the vOT (Fahy et al., 1993; Li et al., 1993; Sobotka and Ringo, 1993; van Turennout et al., 2000; Baker et al., 2002; van Turennout et al., 2003; Freedman et al., 2006; Op de Beeck et al., 2007; Anderson et al., 2008; Op de Beeck et al., 2008; Woloszyn and Sheinberg, 2012; Meyer et al., 2014; Manahova et al., 2018; Koyano et al., 2023). This phenomenon, often referred to as a familiarity effect or familiarity suppression, reflects reduced neural responses to repeatedly encountered visual stimuli (Li et al., 1993; Meyer et al., 2014; Huang et al., 2018). From this perspective, the ND250 may index a domain-general visual familiarity effect arising from neural populations in the vOT/IT cortex. Consistent with this interpretation, a recent study reported that the ND250 was elicited by unfamiliar app icons compared to familiar ones (Hu et al., 2023), suggesting that the ND250 is not exclusively elicited by visual words.

It is important to note that the present findings should not be taken as evidence against the Interactive Account. It remains possible that prediction-error signals, as proposed by this framework, are reflected in other neural responses. Likewise, the present findings should not be interpreted as contradicting predictive coding theories more broadly. In addition to the visual–orthographic familiarity account, the ND250 may also reflect a form of prediction error arising within orthographic processing itself, without requiring phonological or semantic influences, as proposed in predictive coding accounts of visual word recognition (Gagl et al., 2020).

Finally, it should be noted that although non-character components are unpronounceable, many of them carry semantic information and typically function as semantic indicators within the characters they form. For example, the component “扌” is commonly associated with hand-related actions, whereas “钅” often signals a relation to metal. As a result, both character-components and non-character-components may receive top-down semantic predictions during visual processing. For this reason, the present study did not compare group [A] with group [E], or group [B] with group [F], as a critical test of the Interactive Account. Supplementary analyses showed that neither comparison elicited a reliable ND250 effect (see Supplementary Figure S1). This null result is also consistent with the visual-orthographic familiarity account of the ND250, as component frequency was well matched between the compared groups (see Supplementary Table S1).

### 4.2 The functional role of the ND250 in visual word recognition

Reading is a relatively recent cultural invention rather than an evolutionarily inherited biological skill. According to the Neuronal Recycling Hypothesis (Dehaene and Cohen, 2007, 2011), reading relies on the reutilization of pre-existing neural systems that originally evolved for more general visual processing functions. From this perspective, it is reasonable that the ND250 observed during reading reflects a domain-general visual mechanism rather than a language-specific process. An important question, however, concerns the functional relevance of the ND250 for visual word recognition. Does the neural mechanism indexed by the ND250 play an active role in reading, or does it reflect a visual processing mechanism that accompanies reading without directly contributing to it?

Several theoretical perspectives have been proposed to explain the functional significance of the visual familiarity effect in visual object recognition. First, familiarity-related suppression may enhance coding efficiency and reduce metabolic costs by attenuating neural responses to redundant or predictable information (Freedman et al., 2006; Woloszyn and Sheinberg, 2012). Second, reduced responses to familiar stimuli may decrease the salience of expected and thus uninformative events, thereby facilitating the detection of novel or unexpected stimuli (Meyer and Olson, 2011). Third, familiarity-related suppression may place neurons in a state of heightened readiness, enabling more efficient responses to subsequent inputs in dynamic visual environments (Meyer et al., 2014), a mechanism that has been linked to faster processing speed (Manahova et al., 2020). These functional roles are highly relevant to visual word recognition. Specifically, a visual-orthographic familiarity effect may enhance reading efficiency by reducing the cognitive and neural resources required to process highly familiar orthographic forms, thereby enabling rapid reading with reduced neural effort. At the same time, suppression of neural responses to high-frequency words may increase the relative salience of low-frequency or novel words, allowing the visual system to allocate processing resources more effectively during reading.

However, the current evidence does not yet permit a definitive conclusion regarding the functional role of the ND250 in visual word recognition. Further research will be required to clarify whether the ND250 reflects a mechanism that actively contributes to reading efficiency or whether it primarily indexes a general visual process that accompanies reading without being essential to it.

### 4.3 Limitation

A limitation of the present study is that ERPs were recorded under an implicit task, which does not require explicit phonological or semantic processing. Although the characteristics of the ND250 suggest that it is relatively insensitive to task demands, the present findings should be interpreted as reflecting the mechanisms underlying the ND250 under implicit reading conditions. Future studies using explicit tasks may further clarify whether task demands modulate these effects.

## 5 Conclusion

The present study dissociated visual–orthographic familiarity from phonological and semantic associations using Chinese components to clarify the functional basis of the ND250. The results showed that, under implicit task conditions, the ND250 is selectively modulated by the frequency of visual–orthographic forms, rather than by the frequency with which stimuli are associated with phonological or semantic information. These findings challenge the view that the ND250 reflects phonological–semantic top-down prediction acting on orthographic processing, as proposed by the Interactive Account. Instead, they suggest that, under implicit task conditions, the ND250 is more consistent with an electrophysiological marker of visual–orthographic familiarity. More broadly, the results underscore the importance of visual–orthographic familiarity as a foundational factor shaping early neural responses during visual word recognition.

## Data Availability

The raw data supporting the conclusions of this article will be made available by the authors, without undue reservation.
