# Supplementary material for: The ND250 indexes a visual–orthographic familiarity effect in visual word recognition: evidence from Chinese components
Source: Front Psychol. 2026 May 12;17:1783812. doi: 10.3389/fpsyg.2026.1783812 (PMC13201515; doi:10.3389/fpsyg.2026.1783812)
Supplement: Supplementary file 1 [file Data_Sheet_1.pdf]

Supplementary Materials

1. Supplementary Tables

Table S1. Mean log-transformed character frequency and component frequency for each group.

Stimulus group	Character frequency (Mean ± SD)	Component frequency (Mean ± SD)
Group [A]	2.24 ± 0.76	3.73 ± 0.34
Group [B]	2.36 ± 0.66	2.86 ± 0.33
Group [C]	2.90 ± 0.26	3.42 ± 0.53
Group [D]	1.70 ± 0.47	3.17 ± 0.53
Group [E]	0 ± 0	3.79 ± 0.33
Group [F]	0 ± 0	2.69 ± 0.39

Table S2. Behavioral results.

Stimulus group	Mean accuracy rate ± SD (%)	Mean response time ± SD (ms)
Group [A]	97.40 ± 2.04	475.16 ± 67.91
Group [B]	96.62 ± 2.36	478.01 ± 67.82
Group [C]	96.67 ± 2.41	477.95 ± 66.98
Group [D]	97.23 ± 1.85	474.86 ± 67.67
Group [E]	96.38 ± 1.83	474.86 ± 63.67
Group [F]	96.96 ± 1.75	474.92 ± 64.09

2. Supplementary Figures

Figure S1 presents the results of the mass univariate ERP analyses across stimulus groups. No significant ND250 effect was observed for the comparisons between group [A] and group [E], or between group [B] and group [F]. Notably, a significant ERP difference between group [A] and group [E] was observed at early latencies (i.e., before 200 ms). This early effect is attributable to the fact that the N170 elicited by group [A] peaked slightly earlier than that elicited by group [E] (see Figure S2). Such early ERP differences are likely related to differences in low-level visual properties across stimuli, as individual Chinese components differ in their visual features.

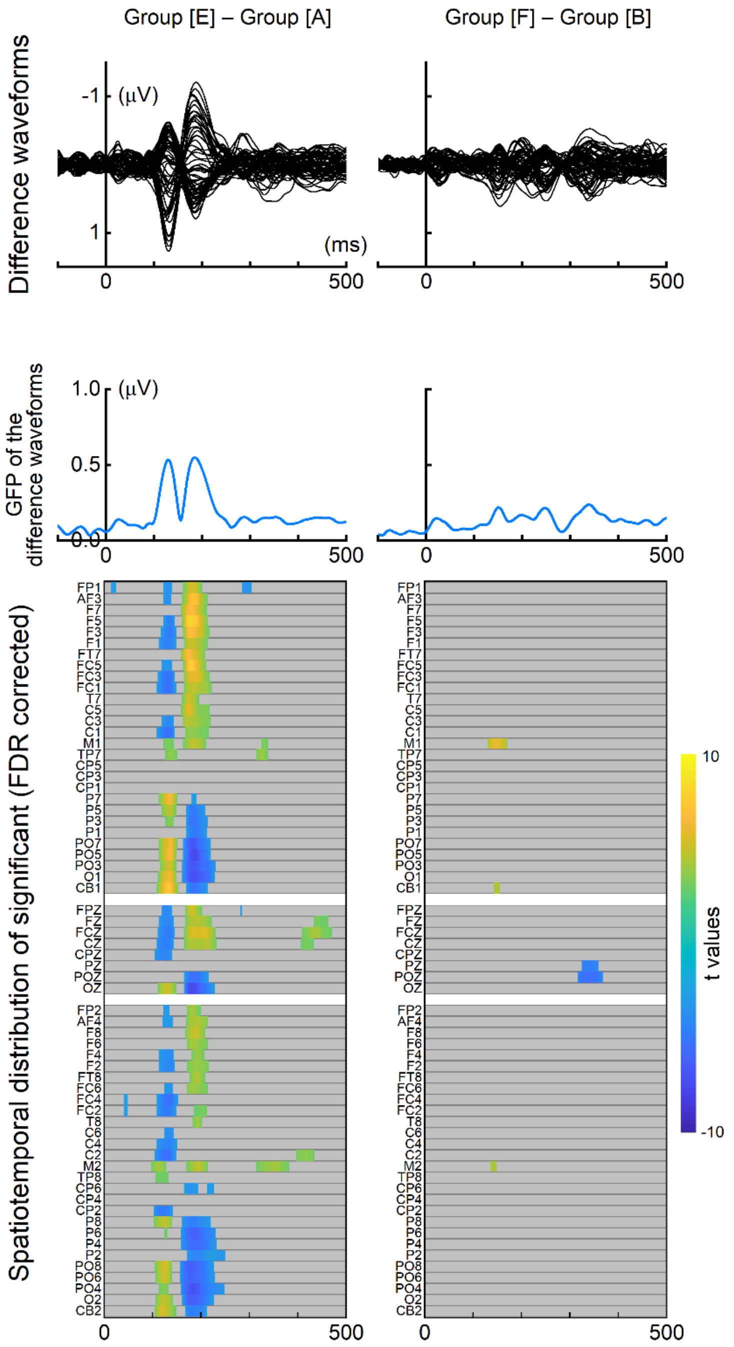

Figure S1. ERP difference between stimulus groups. ERPs elicited by one stimulus group were subtracted from those elicited by the other group at each of the 64 recording electrodes. The resulting grand-averaged difference waveforms are shown in the top panels. The global field power (GFP) of the difference waveforms for each comparison is displayed in the middle panels. Repeated-measures, two-tailed *t* tests were conducted at each time point between 50 and 500 ms post-stimulus onset. The resulting spatiotemporal distribution of significant effects, corrected for multiple comparisons using the FDR procedure, is shown in the bottom panels. Non-significant time points are masked in gray, whereas significant time points are visualized using a heatmap, with color intensity representing the corresponding *t* values.

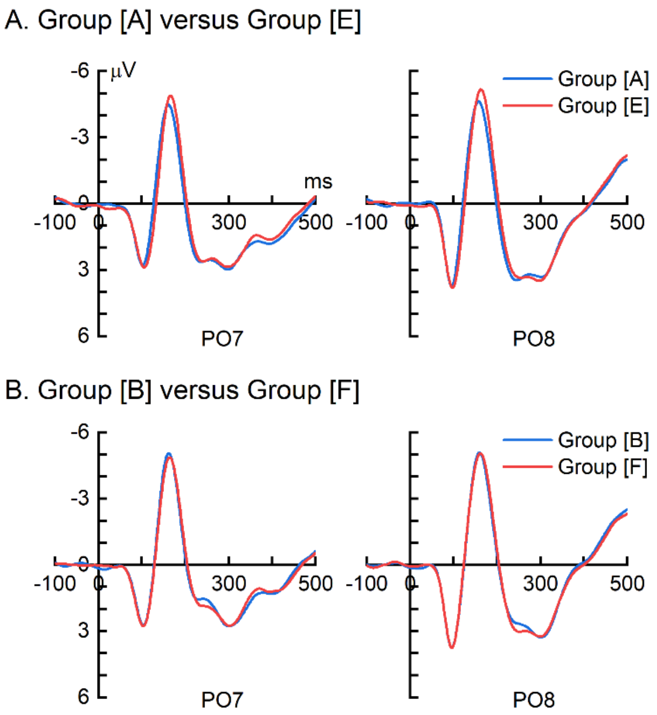

Figure S2. Grand-averaged ERPs recorded at electrodes PO7 and PO8. (A) Comparison of ERPs elicited by group [A] and group [E]. (B) Comparison of ERPs elicited by group [B] and group [F].
